# Silver Needle Thermal Therapy Improves Mitochondrial Injury in the Skeletal Muscle of MPS Rats by Inhibiting the TRPV1/CaMKII Pathway

**DOI:** 10.33549/physiolres.935508

**Published:** 2025-06-01

**Authors:** Yuanyue HUANG, Songsong AN, Yangxiaoyu TANG, Xingyue YANG, Changyu SHEN, Yuanxin HUANG, Lin WANG, Chunxin WO

**Affiliations:** 1 Guizhou Medical University, Guiyang, China; 2 Pain Department, The Affiliated Hospital of Guizhou Medical University, Guiyang, China

**Keywords:** SNT, MPS, Mitochondria, TRPV1, CaMKII

## Abstract

The objective of this study is to elucidate the therapeutic mechanisms underlying silver needle thermal therapy (SNT) in alleviating skeletal muscle mitochondrial damage in a rat model of myofascial pain syndrome (MPS), with particular emphasis on its regulatory role concerning TRPV1/CaMKII. The MPS rat model was established through blunt impact and eccentric movement. Interventions included SNT and local intramuscular injections of anti-TRPV1 miRNA. Behavioral assessments were conducted to measure the mechanical and thermal pain thresholds of the rats. Histopathological staining was performed to evaluate muscle structure, while mitochondrial damage was assessed using transmission electron microscopy. Western blotting analysis was employed to quantify expression levels of TRPV1, CaMKII, and CytC. Additionally, immunofluorescence techniques were applied to analyze both the expression levels of TRPV1 and its co-localization with CaMKII. Following administration of SNT and anti-TRPV1 miRNA injections, a downregulation in the expression levels of TRPV1, CaMKII, and CytC within the muscle tissue of MPS rats was observed; concurrently, mitochondrial damage exhibited improvement. The implementation of SNT and the inhibition of TRPV1 lead to a reduction in CaMKII, thereby alleviating mitochondrial damage, indicating that TRPV1 is a potential target for silver needle thermal therapy of MPS.

## Introduction

Myofascial pain syndrome (MPS), an aseptic inflammation of skeletal muscle, is characterized by myofascial trigger points (MTrPs) as the main clinical manifestation. The persistent contractile activity of MTrPs can cause local ischemia, hypoxia, and neurophysiological alterations of nociceptors, leading to pain sensitization and referred pain [1]. Myofascial pain syndrome is a common disease, with a prevalence of approximately 30 % to 85 % among patients with musculoskeletal pain [2]. Owing to the incomplete understanding of the pathogenesis, patients with myofascial pain syndrome are prone to missed diagnosis and progression to intractable chronic pain, which seriously affects the quality of life of these patients. Previous studies have revealed that mitochondrial damage in skeletal muscle participates in the pathogenesis of myofascial pain syndrome [3].

Mitochondria serve as the energy production units within skeletal muscle and are the primary organelles responsible for generating ATP energy through respiration and oxidative phosphorylation. They are also significant participants in the pain response and can modulate the duration of harmful signal transmission [4]. Changes in both the quantity and functionality of mitochondria can result in alterations in pain-related behaviors, thereby facilitating the pathogenesis of various types of pain [5]. In MPS, mitochondrial dysfunction gives rise to disorders in energy metabolism, further intensifying the ischemic and hypoxic conditions of the muscles [6,7]. Consequently, mitochondrial damage is not merely a pathological consequence of MPS but also a crucial driving factor for its persistent pain.

Silver needle thermal therapy (SNT) bears resemblance to traditional acupuncture yet exhibits certain distinctions. Silver needles are positioned within muscles, tendons, and fascia rather than at acupoints, and a specialized machine is employed to heat the needles for the purpose of eliminating aseptic inflammation and alleviating pain. The pain-alleviating effect of the silver needle therapy mainly stems from three mechanisms: eradicating aseptic inflammation, enhancing blood circulation, and relieving muscle spasms [8]. Research has confirmed that the temperature of the silver needle within the body can reach 41 °C or above, and the conducted heat energy gives rise to complex biological effects, exerting favorable efficacy in the treatment of intractable soft tissue pain [9]. It is noteworthy that the high-temperature properties of SNT might function through activating or regulating certain ion channels, particularly the transient receptor potential vanilloid 1 (TRPV1) channel related to pain.

Transient receptor potential vanilloid 1 (TRPV1), a non-selective cation channel abundantly expressed in nociceptors, triggers intracellular Ca^2+^ influx upon activation, thereby activating downstream signaling pathways [10] and playing a crucial role in the development of pain. TRPV1 can be activated by high temperatures (≥40 °C) and capsaicin, among others [11], while the temperature of silver needles *in vivo* can exceed 41 °C, enabling the activation of TRPV1 and potentially resulting in TRPV1 desensitization after prolonged activation. Research indicates that TRPV1 is localized in the sarcoplasmic reticulum. Once activated, it facilitates the release of Ca^2+^ from the sarcoplasmic reticulum into the cytoplasm [12], subsequently resulting in mitochondrial Ca^2+^ overload and mitochondrial dysfunction [4]. The expression of TRPV1 is upregulated in various pain-related diseases, and reducing its expression can suppress the maintenance of chronic pain [13–15]. TRPV1 is not merely a crucial molecule in the transmission of pain signals but may also be a significant bridge linking mitochondrial dysfunction to the pain mechanism of MPS.

CaMKII (Calmodulin-dependent kinase II) functions as a sensor protein that becomes activated in the presence of Ca^2+^ and calmodulin (CaM) [16]. It serves as a crucial effector protein within the mitochondrial apoptotic pathway that is triggered by alterations in intracellular Ca^2+^ levels. It might induce the loss of mitochondrial membrane potential and subsequent dysfunction by directly acting on the mitochondrial membrane and mediate the release of cytochrome c (CytC) from mitochondria [17]. A research indicates that inhibiting the expression of TRPV1 in the spinal cord can downregulate the expression of CaMKII to facilitate the alleviation of neuropathic pain [18]. There exists a close molecular crosstalk between TRPV1 and CaMKII, particularly in the processes of mitochondrial dysfunction and pain maintenance.

## Materials and Methods

### Animal

SPF healthy adult male SD (Sprague Dawley) rats (~200 g) were obtained from Guizhou Medical University’s Animal Experiment Center. They were housed in a clean, ventilated room with 50 % humidity, constant temperature (22.0±0.5) °C, and a 12-hour light/dark cycle (7:00 AM to 7:00 PM), with ample food, water, and regular bedding changes. The study was approved by the university’s Animal Ethics Committee (approval number: 2201546), and efforts were made to minimize animal use and suffering.

### Model building

The myofascial pain syndrome (MPS) rat model was established using Huang Qiangmin’s method [19]. Three days before modeling, rats underwent treadmill adaptation. On day 1, rats were anesthetized with 1 % pentobarbital sodium (3–4 mg/100 g) and subjected to a 2.352 J impact on the right vastus medialis. On days 2 and 3, rats ran downhill at 16 m/min for 90 min at −16 °C, driven by auditory and electrical cues. Rats rested for the next 4 days. This intervention was repeated weekly for 8 weeks, followed by 4 weeks of normal feeding. After recovery, 4 rats were randomly selected to check for hard nodules or tense bands in the right vastus medialis, with tissue samples taken for hematoxylin-eosin staining to confirm successful modeling.

### In vivo RNA interference

At week 9 post-MPS model establishment, AAV9-TRPV1-RNAi (Shanghai Jikaigene Technology Co., LTD) was injected intramuscularly into the right vastus medialis at three sites (15 μl total). Behavioral tests were performed before (8 weeks) and after transfection (12 weeks), with TRPV1 expression levels confirmed by Western blot to validate transfection efficacy.

### Silver needle thermal therapy

At 12 weeks post-modeling, SNT was performed. Rats were anesthetized and secured supine. A clinician palpated the right vastus medialis to identify tension bands or nodules, marking the treatment area. After disinfection, two sterile silver needles (0.5 mm×10 cm) were inserted parallel to muscle fibers (1 cm apart) through the tension band to myofascial bone attachment points. Using a silver needle thermal conduction instrument (Shanghai Shuxin Technology, YRX160256), needles were heated to 110 °C, with skin temperature reaching 43 °C, for 15 min. Post-treatment, rats were returned to cages for normal care.

### Behavioral assessment

Mechanical and thermal pain thresholds were evaluated at three distinct time points: prior to model induction (0 weeks), during the modeling phase (4 weeks), and post-modeling (8 weeks). At week 9, anti-TRPV1 miRNA was administered *via* local injection, and pain thresholds were reassessed at week 12. Subsequently, silver needle thermal therapy (SNT) was conducted, followed by a final assessment of pain thresholds at week 14. For behavioral assessments, rats were acclimatized in a metal mesh cage for 10–20 min prior to testing. Mechanical pain sensitivity was determined using Von Frey filaments applied to the mid-plantar region of the right hind paw for 6–8 s. Responses were categorized as “O” (no response) or “X” (paw withdrawal or licking), and the mechanical pain threshold was calculated based on the sequence of responses. Thermal pain sensitivity was measured using a PL-200 thermal nociception apparatus. Rats were positioned on a glass platform, and the heat source was directed at the mid-plantar area of the right hind paw. Three measurements were taken at 5-minute intervals, with a maximum cutoff time of 20 s to prevent tissue injury. The mean value of the three measurements was recorded as the thermal pain threshold. Throughout the testing process, observers were blinded to the experimental group assignments to ensure unbiased data collection.

### Hematoxylin-eosin staining

Rats were euthanized through an intraperitoneal injection of a 1 % sodium pentobarbital solution. Subsequently, the heart was perfused with physiological saline, and samples were obtained from the right vastus medialis. These specimens were fixed in 4 % paraformal-dehyde for 24 h and subsequently dehydrated using ascending concentrations of ethanol prior to being cleared in xylene. After embedding in paraffin, sections measuring 5 μm were cut and underwent deparaffinization in xylene. The sections were then rehydrated with descending concentrations of ethanol and stained with hematoxylin and eosin staining solution before proceeding to immunostaining. The morphology and arrangement of muscle fibers were observed under a light microscope.

### Transmission electron microscopy

Taking a 1 mm^3^ sample of the muscle from the right vastus medialis, it was fixed with 2.5 % glutaraldehyde at 4 °C overnight. The samples were then washed three times with phosphate-buffered saline (PBS), each wash lasting for 15 min. Subsequently, the tissues were fixed in 1 % osmium tetroxide for 2 h at room temperature and rinsed three times with PBS for 15 min each time. Following fixation, samples underwent dehydration using increasing concentrations of ethanol solutions and acetone. After embedding in epoxy resin, they were placed in an oven to undergo polymerization. Ultra-thin sections were prepared using a Leica UC6 ultramicrotome and double-stained with uranyl acetate and lead citrate at a concentration of 3 %. Finally, the samples were examined and photographed using transmission electron microscopy (JEM-1400 FLASH, JEOL) operated at an accelerating voltage of 110 kV.

### Determination of ATP content

The ATP level in the right vastus medialis was measured using a firefly luciferase-based ATP detection kit (S0026, Beyotime). Tissue was lysed with ATP lysis buffer, centrifuged, and the supernatant was collected. Protein concentration was determined using the BCA kit (ZJ102, Epizyme). The supernatant was mixed with ATP detection solution, and luminescence was measured with a microplate reader (Stbergy H4, Bio Tek). ATP concentration was calculated using the ATP standard curve.

### Immunofluorescence

Right vastus medialis tissue samples were fixed in 4 % paraformaldehyde at 4 °C for three days, cryoprotected in 30 % sucrose overnight at 4 °C, embedded in OCT medium, snap-frozen in liquid nitrogen, and stored at −80 °C. Sections (5 μm) were cut at −20 °C, placed on slides, and treated with 10 % bovine serum for one hour. Samples were incubated overnight at 4 °C with primary antibodies (TRPV1, FHIL1, or CaMKII at 1:10 dilution in 10 % bovine serum albumin), followed by two-hour incubation at room temperature with secondary antibodies (Alexa Fluor®488 donkey anti-rabbit IgG and Alexa Fluor®594 donkey anti-goat IgG, both at 1:500). Sections were mounted with DAPI-containing medium and visualized using a fluorescence microscope (ZEISS, Axio Imager).

### Western blotting

Right vastus medialis tissue from rats was stored in a freezer at −80 °C. For analysis, tissue was weighed, cut, and homogenized in RIPA lysis buffer, then incubated on ice for 30 min. After centrifugation, the supernatant was used for protein quantification *via* the BCA method. Protein samples were denatured, separated by SDS-PAGE (Sodium Dodecyl Sulfate Polyacrylamide Gel Electrophoresis), and transferred to a PVDF membrane. The membrane was washed, blocked for 1 h, and incubated overnight at 4 °C with primary antibodies: TRPV1 (1:2000), CaMKII (1:1000), CytC (1:4000), and GAPDH (1:1000) All antibodies utilized in this study were acquired from Proteintech. After washing, secondary antibodies (HRP-conjugated Goat Anti-Rabbit IgG and HRP-conjugated Goat Anti-Mouse IgG, 1:5000) were applied for 60 min at room temperature. The membrane was developed using a chemiluminescence substrate kit and a chemiluminescence imaging system (CLINX, Chemi Scope6200). Image J software (Bethesda SNTa, MD USA) was employed to quantify the intensity of protein bands detected on the blots.

### Statistical analysis

Statistical analysis was conducted using SPSS Statistics and GraphPad Prism software. Data are presented as mean ± standard error of the mean (SEM). Differences between groups were evaluated using one-way (ANOVA) followed by *post hoc* Tukey’s test. P<0.05 was considered to indicate a statistically significant difference.

## Results

### Mechanical hyperalgesia and thermal hyperalgesia in MPS rats

Myofascial pain was successfully induced in MPS rats through striking and eccentric exercise. After 4 weeks of the MPS model, the rats exhibited reduced mechanical and thermal pain thresholds in the ipsilateral hind paw when compared to normal rats, with a more pronounced decrease observed after 8 weeks of modeling. The results indicated that the rats displayed increased sensitivity to both mechanical and thermal stimuli.

**Fig. 1:**
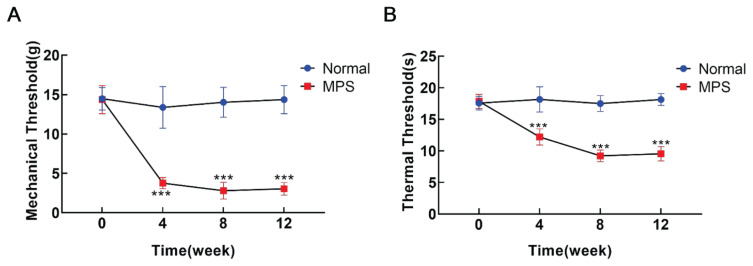
Mechanical withdrawal threshold (MWT) and paw withdrawal thermal latency (PWTL) respectively stand for mechanical threshold (gram, g) and thermal threshold (second, s) during modeling in rats. (**A**) Four weeks post-modeling, the rats exhibited pronounced mechanical hyperalgesia, indicated by a significant reduction in the paw withdrawal threshold of the right hind paw to mechanical stimulation. After eight weeks, this threshold further declined and stabilized at twelve weeks. (**B**) Notable thermal hyperalgesia was observed eight weeks after modeling, as evidenced by a decrease in paw withdrawal latency for the right hind paw. This thermal hyperalgesia threshold reached its lowest point at eight weeks and remained stable thereafter at twelve weeks. Data are expressed as mean ± SEM, with error bars representing SEM. *** P<0.001 compared to control group (ANOVA; n=10 rats per group).

Furthermore, the decline in mechanical and thermal pain thresholds appeared to stabilize following 12 weeks of modeling (Fig. 1A, B).

### The expression of TRPV1 in the muscles of MPS rats was increased

**Fig. 2:**
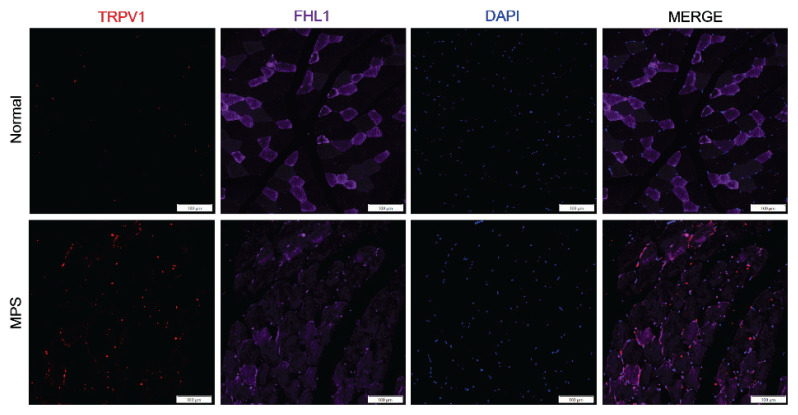
Immunofluorescence staining demonstrating the expression of transient receptor potential V1 (TRPV1) protein in the right vastus medialis of rats. The expression level of TRPV1 (red) was significantly elevated in MPS-treated rats. Scale bar represents 100 μm.

To investigate the potential causes of hyperalgesia, we observed that TRPV1 expression was elevated in the right vastus medialis of MPS rats compared to normal rats. FHL1 serves as a marker for skeletal muscle cells (Fig. 2).

### Silver needle thermal therapy reduced TRPV1-dependent mechanical hyperalgesia

**Fig. 3:**
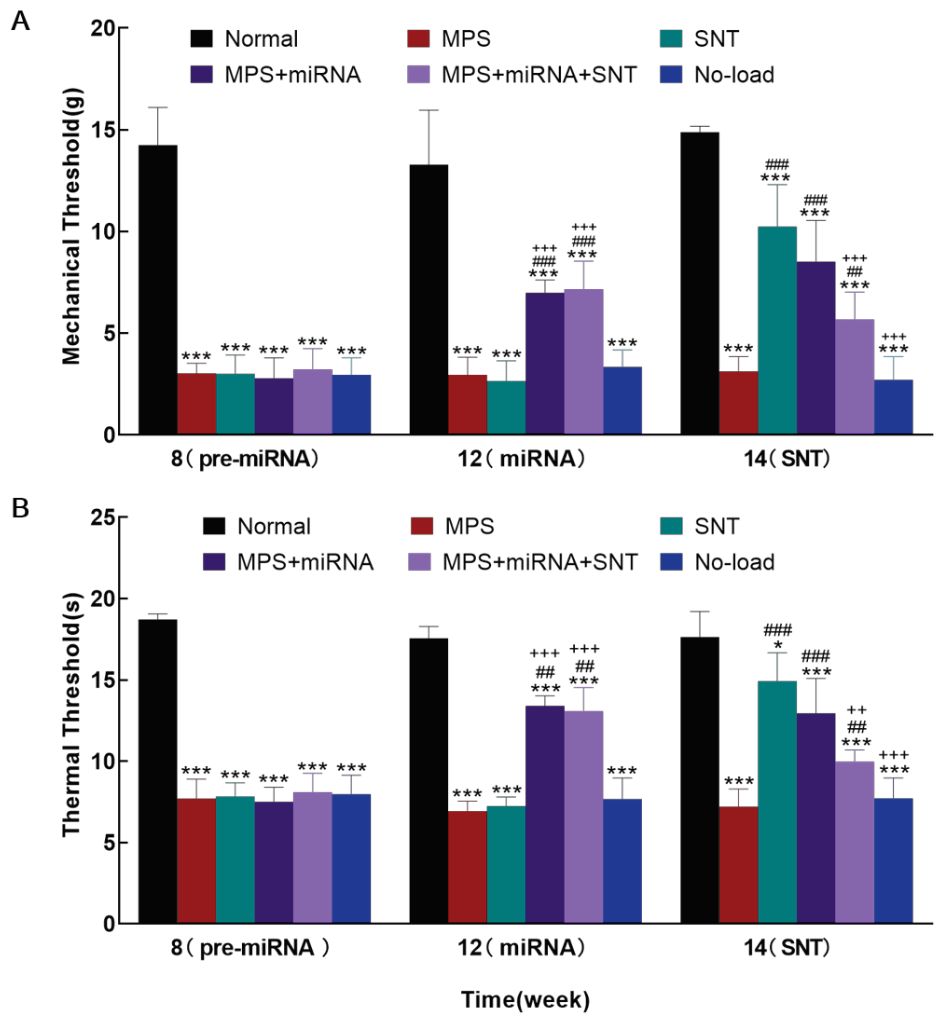
Mechanical paw withdrawal thresholds and thermal latencies in six groups of rats. The six groups included: Normal group, MPS (myofascial pain), SNT (myofascial pain with SNT), MPS+miRNA (myofascial pain with TRPV1-related miRNA transfection), MPS+miRNA+ SNT (myofascial pain with TRPV1-related miRNA transfection and SNT), and No-load (myofascial pain accompanied by adenovirus empty load). (**A**) Mechanical hyperalgesia was assessed using the von Frey test across the six groups. (**B**) Thermal hyperalgesia was evaluated through the Hargreaves test among the same groups. Comparisons to the Normal group at equivalent time points revealed * P<0.05, *** P<0.001; comparisons to the MPS group at equivalent time points revealed ^##^ P<0.01, ^###^ P<0.001; comparisons to the SNT group at equivalent time points revealed ^++^ P<0.01, ^+++^ P<0.001; (ANOVA; n=6 rats per group).

We administered anti-TRPV1 miRNA to the local muscles to downregulate TRPV1 protein expression, and subsequently measured mechanical and thermal pain thresholds to investigate the role of TRPV1 in myofascial pain maintenance. Rats transfected with anti-TRPV1 miRNA (12 weeks) demonstrated increased mechanical (Fig. 3A) and thermal pain thresholds (Fig. 3B) compared to those MPS rats. Following silver needle thermal therapy (14 weeks), the SNT group exhibited significantly higher mechanical (Fig. 3A) and thermal (Fig. 3B) pain thresholds than the MPS group; however, in the MPS+miRNA+SNT group, these thresholds were lower than those observed in both the SNT group and the MPS+miRNA group (Fig. 3A, B).

### Silver needle thermal therapy enhances muscle structure in MPS rats

**Fig. 4:**
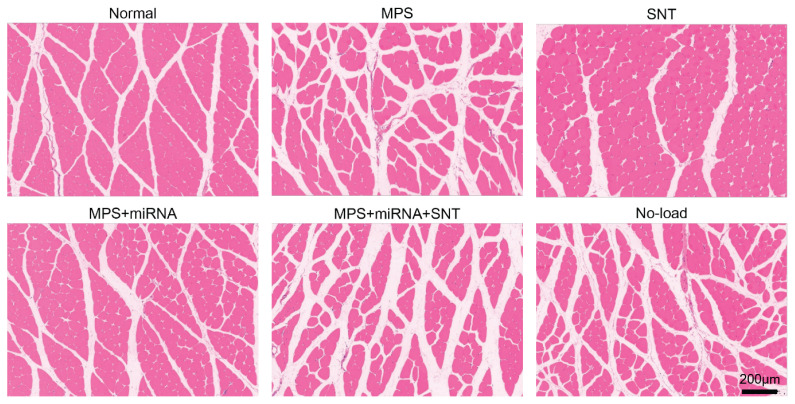
Extract partial tissues from the right vastus medialis of six groups of rats, optical microscopic images of cross-sections of muscle fibers stained with H&E. In the normal group, muscle cells present as uniformly sized polygons. In the MPS group, several muscle cells of various sizes and shapes similar to circles emerged. Abnormal fibers clustered together, showing short cracks and large gaps within the framework of muscle fibers. The scale bar is 200 μm.

The muscle cells in the normal group exhibited regular, round or polygonal shapes, demonstrating uniform size and a closely arranged structure. In contrast, the MPS group displayed atrophic and degenerated muscle fibers; oval and round muscle cells of varying sizes were observed alongside irregularly widened intercellular spaces, with some instances of nuclear migration noted. The SNT group showed improvement in muscular structural disorder, with the morphology of muscle cells approximating normalcy. Meanwhile, the morphology of muscle cells in the MPS+miRNA group was also found to be near-normal. The muscular structure within the MPS+miRNA+SNT group demonstrated enhancements compared to that of the MPS group; however, it remained inferior to both the structures observed in the MPS+miRNA and SNT groups. The muscle structure disorder of the No-load group tended to be analogous to that of the MPS group (Fig. 4).

### Silver needle thermal therapy reduced mitochondrial damage in MPS rats

**Fig. 5:**
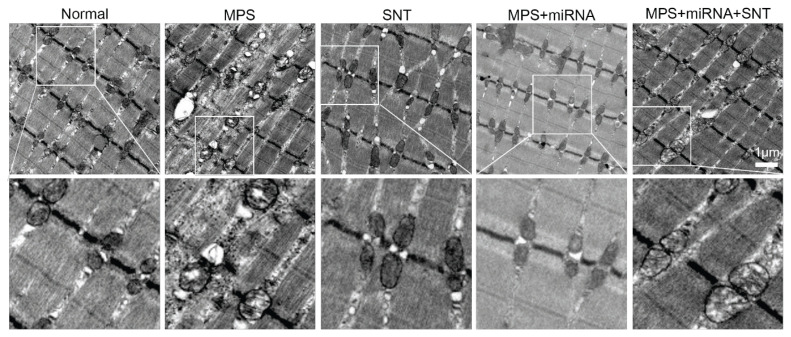
Representative electron microscopic images of the distribution and morphology of muscle mitochondria in the right vastus medialis of six groups of rats. The morphology of mitochondria in the MPS group is typically significantly different from that of the normal group. In the muscle fibers of the MPS group, the size of mitochondria is usually larger. The size and morphology of mitochondria in the MP+miRNA+SNT group are more similar to those of the MPS group. While the size and morphology of mitochondria in the SNT group and the MPS+miRNA group are closer to those of the normal group. The scale bar is 1 μm.

In the normal group, skeletal muscle mitochondria exhibited an oval shape, while the outer mitochondrial membrane remained intact. The matrix was uniform, and the cristae were elongated and oriented perpendicular to the long axis of the mitochondria. Additionally, skeletal muscle myofilaments were neatly arranged. In contrast, in the MPS group, mitochondria within the muscle tissue appeared swollen; cristae were fragmented or absent, lamellae were reduced in number, and the parallel structure of residual ridges had disappeared. This was accompanied by localized focal lysis that displayed vacuole-like changes. In the SNT group, mitochondrial swelling in muscle tissue showed a significant reduction or approached normalcy; furthermore, lamellar bodies were markedly increased. The mitochondrial architecture in muscular tissues within the MPS+miRNA group also tended toward normalization. However, in the MPS+miRNA+SNT group, while partial relief from mitochondrial swelling was observed, disorganization of ridge arrangement persisted (Fig. 5). Our research findings suggest that SNT significantly ameliorated the structural damage of skeletal muscle mitochondria in MPS rats. Further exploration of the structural effects of antagonizing TRPV1 on skeletal muscle mitochondria in MPS rats revealed that the damaged mitochondria were also improved to a large extent. Meanwhile, when SNT and antagonizing TRPV1 were performed simultaneously, the degree of mitochondrial repair was relatively weak, fully indicating that the improvement of mitochondrial dysfunction by SNT is likely to exert its effect through the TRPV1 channel.

### The expression of TRPV1, CaMKII, and CytC decreased after silver needle thermal therapy, and augmented the content of ATP

**Fig. 6:**
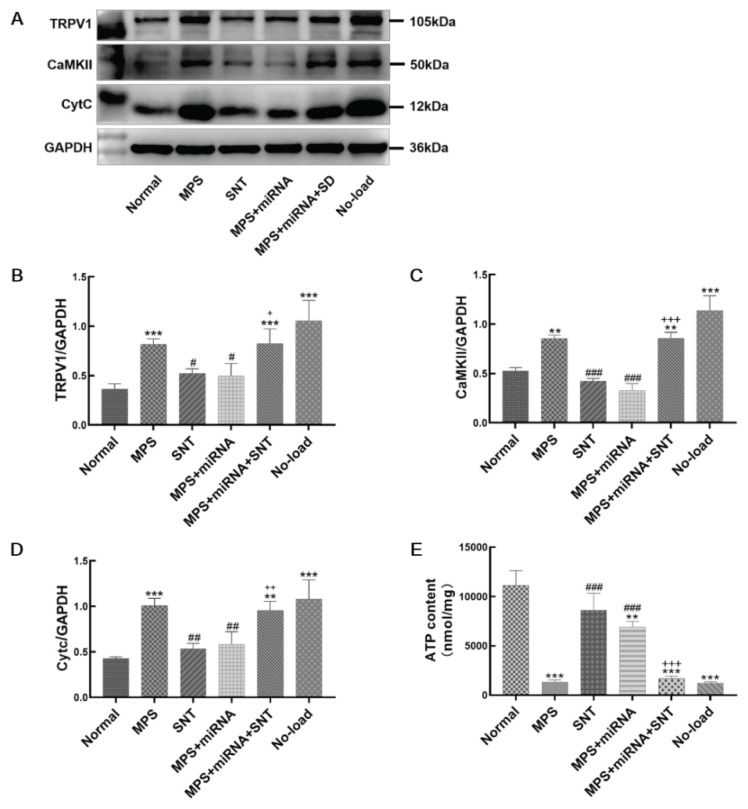
Expression levels of transient receptor potential V1 (TRPV1) and related molecules in the right vastus medialis of rats. (**A**) Immunoblot images depict the six lanes representing the target proteins in the following order: Normal, MPS, SNT, MPS+miRNA, MPS+miRNA+SNT, and No-load groups. (**B**) Changes in TRPV1 protein expression. (**C**) Changes in CaMKII protein expression. (**D**) Changes in CytC protein expression. (**E**) The bar graph describes the ATP content in each group. ** P<0.01, *** P<0.001 indicate significant statistical differences when compared with the normal group. ^#^ P<0.05, ^##^ P<0.01, and ^###^ P<0.001 indicate statistical differences relative to the MPS group. ^+^ P<0.05, ^++^ P<0.01; ^+++^ P<0.001 indicate significant differences when comparing against the SNT group.

The expression levels of TRPV1, CaMKII, and CytC in skeletal muscle were assessed using Western blotting, and the ATP levels in the skeletal muscle were measured through ATP detection kits. The results indicated that the expression levels of TRPV1 (Fig. 6A, B), CaMKII (Fig. 6A, C), and CytC (Fig. 6A, D) were significantly elevated, and the ATP (Fig. 6E) content was reduced in MPS rats. These alterations were reverse in both the SNT and MPS+miRNA groups, with a lesser degree of attenuation observed in the MPS+miRNA+SNT group (Fig. 6A, B, C, D). The increased expression of CytC and the reduced content of ATP act as indicators of mitochondrial damage. These findings suggest that SNT inhibits the expression of TRPV1 and CaMKII, thereby reducing mitochondrial injury in MPS rats. However, levels of TRPV1, CaMKII, and CytC within the MPS+miRNA+SNT group remained higher than those found in the SNT group (Fig. 6A, B, C, D), the ATP content was lower than that in the SNT group. This observation indicates that when TRPV1 is antagonized, the therapeutic efficacy of silver acupuncture may be diminished. This finding further corroborates that SNT exerts its effects primarily through inhibition of TRPV1.

### Colocalization of TRPV1 and CaMKII in skeletal muscle

**Fig. 7:**
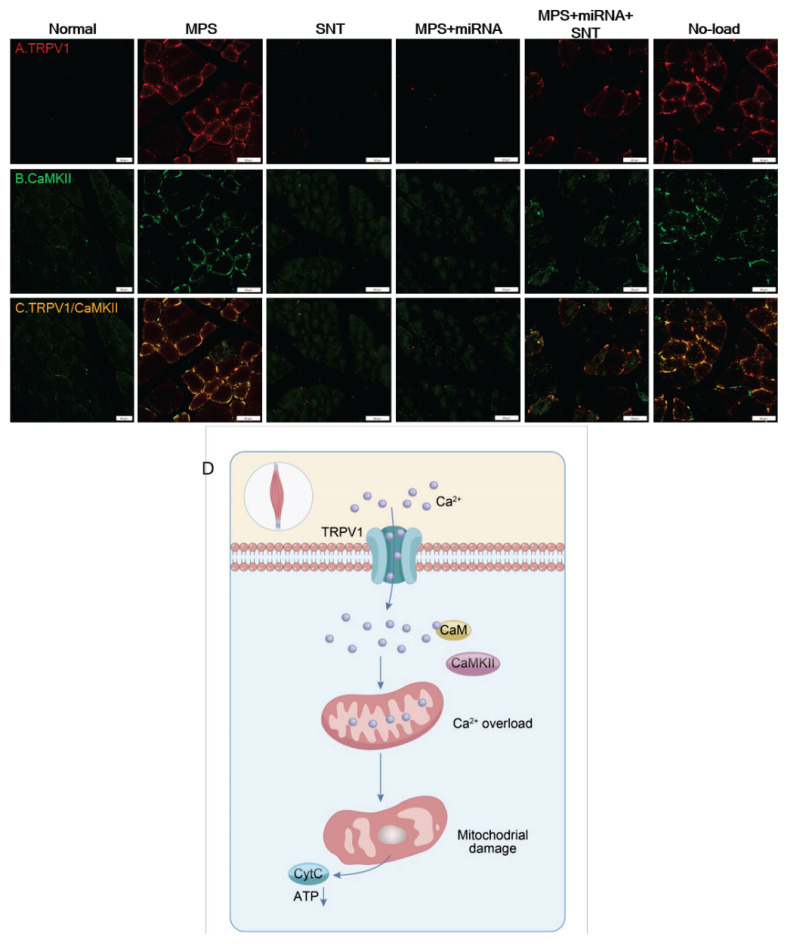
Immunofluorescence staining of transient receptor potential V1 (TRPV1) and CaMKII protein expression in the right vastus medialis of rats. (**A**) The expression level of TRPV1 (red) is significantly elevated in the skeletal muscle of MPS rats. (**B**) A notable increase in CaMKII (green) expression was observed in the skeletal muscle of MPS rats. (**C**) Co-localization of TRPV1 and CaMKII is markedly enhanced, appearing orange, within the skeletal muscle of MPS rats. Scale bars represent 50 μm. (**D**) Working model for the role of TRPV1 and CaMKII in the control of mitochondrial damage.

The immunofluorescence images of the muscle revealed similarities in TRPV1 immunoexpression to the alterations measured by Western blotting. Specifically, there was an increase in TRPV1 expression in the MPS rat model and a significant decrease in expression levels observed within both the SNT and MPS+miRNA groups when compared to normal rats (Fig. 7A).

Furthermore, co-localization of TRPV1 and CaMKII was evident in MPS rats (Fig. 7C), suggesting that the elevation of CaMKII levels is mediated by enhanced TRPV1 expression. Notably, this upregulation was reversed in rats from the SNT and MPS+miRNA groups (Fig. 7C). Based on this, it is easy to infer that TRPV1 may cause mitochondrial damage by regulating the expression of CaMKII (Fig. 7D).

## Discussion

In the present study, we provide evidence that hyperalgesia and the analgesic effects of silver needles in MPS rats are contingent upon the modulation of TRPV1 channels within muscle tissue.

MPS rats exhibited hyperalgesia four weeks post-model establishment, as demonstrated by increased sensitivity to von Frey filaments and a reduction in thermal paw withdrawal latency. These alterations correlate with an upregulation of TRPV1 channel expression, while SNT is associated with a decrease in TRPV1 levels. Collectively, these findings imply that TRPV1 signaling within muscle tissue is a significant contributor to hyperalgesia associated with myofascial pain and represents a primary target for thermal conduction therapy utilizing silver needles.

To date, the molecular mechanism through which SNT regulates myofascial pain syndrome remains inadequately elucidated. Various studies have indicated that in the pathological progression of myofascial pain syndrome, an energy crisis induced by reduced ATP levels can lead to muscle contracture. This subsequent capillary compression creates a hypoxic environment and impairs mitochondrial function, thereby establishing a detrimental cycle [6]. Mitochondria possess a distinctive double-membrane structure. The inner membrane folds inward to form cristae, which significantly enhances the surface area of the inner membrane. The cristae are rich in enzyme complexes involved in oxidative phosphorylation, such as CytC oxidase, and serve as the crucial sites for the electron transport chain and ATP synthesis [20]. A normal mitochondrial membrane structure is capable of maintaining the proton gradient and ensuring the efficient generation of energy during the process of oxidative phosphorylation. When the mitochondrial structure is impaired, for instance, when cristae rupture or membrane integrity is disrupted, it causes the release of CytC, interrupts the continuity of the electron transport chain, increases proton leakage, and leads to a marked reduction in ATP production [21]. Research indicates that in multiple muscle disease models, alterations in mitochondrial structure are invariably accompanied by disorders in energy metabolism [6], further validating the close association between the integrity of the mitochondrial structure and its function. The electron microscopy findings in the research indicated that the skeletal muscle mitochondria of MPS rats exhibited distinct damage manifestations, including obvious swelling, fractured or vanished cristae, and reduced lamellae. By contrast, the mitochondrial structure of MPS rats that underwent SNT and TRPV1 antagonism demonstrated remarkable improvement. The outer mitochondrial membranes were more intact, the matrix was more homogeneous, and the arrangement of cristae was more orderly. Simultaneously, the skeletal muscle of MPS rats was accompanied by increased expression of CytC and decreased ATP content; after SNT treatment and TRPV1 antagonism, the expression of CytC decreased and the ATP content increased. These findings provide compelling evidence for the reparative effects of SNT on skeletal muscle mitochondrial damage, suggesting that this restoration may occur through a reduction in TRPV1 expression.

The damage to mitochondria is linked to the activation of TRPV1 and the subsequent overexpression of CaMKII. TRPV1 is localized in the endoplasmic reticulum of skeletal muscle cells, and its activation facilitates the release of Ca^2+^ from the sarcoplasmic reticulum into the cytoplasm [22]. Ca^2+^ is a prevalently existing second messenger in eukaryotic cells, and it is involved in regulating nearly all cellular behaviors, including proliferation, differentiation, protein synthesis, gene expression and mitochondrial function [23]. One key function of mitochondria is to buffer calcium overload in the cytoplasm The activation of TRPV1 increases mitochondrial Ca^2+^ levels, leading to a disturbance in mitochondrial Ca^2+^ homeostasis, which disrupts mitochondrial membrane potential, induces CytC release, and ultimately results in apoptosis [12]. In this context, CaMKII serves as an important effector protein activated by elevated intracellular Ca^2+^, playing a critical role in the mitochondrial apoptosis pathway triggered by alterations in intracellular calcium concentrations. Through the detection of skeletal muscle samples, it was found that when the amount of Ca^2+^ in skeletal muscle increased, it would be accompanied by the activation of CaMKII, and both presented consistency in terms of time and change trend [24]. This further substantiated the inseparable relationship between the increase in Ca^2+^ and the activation of CaMKII. Recent studies have identified molecular crosstalk between TRPV1 and CaMKII that contributes to pain development and maintenance [18]. In the present study, it was observed that the expressions of TRPV1 and CaMKII were conspicuously augmented in MPS rats, along with mitochondrial damage. Ca^2+^, serving as the mediator linking TRPV1 and CaMKII, occurred concurrently with the activation of CaMKII. The mitochondrial calcium overload triggered by the massive influx of Ca^2+^ following the activation of TRPV1 is highly likely to be the reason for mitochondrial injury in MPS rats.

Silver needle thermal therapy can activate the TRPV1 channel through high-temperature stimulation. In the present study, however, a reduction in TRPV1 expression was witnessed after SNT. This could potentially be ascribed to the prolonged existence of activating stimuli, which causes channel closure and subsequent desensitization of TRPV1 [25]. Channel desensitization represents a fundamental strategy for managing chronic pain, potentially reducing or even eliminating discomfort [26]. Regarding the mechanism of TRPV1 channel desensitization, the widely recognized one is calcium-dependent desensitization. According to the duration of exposure to the activating stimulus and the external calcium concentration, the Ca^2+^ inflow through the TRPV1 channel will desensitize the channel itself. From a cellular perspective, this represents a feedback mechanism that protects nociceptive neurons from toxic Ca^2+^ overload [27], ultimately causing structural rearrangement within the TRPV1 channel complex and resulting in its acute desensitization [28]. Another possible mechanism is heat desensitization. SNT provides a prolonged period of high temperature sufficient to activate TRPV1. After heat-induced activation, if the harmful heat persists, TRPV1 will rapidly desensitize on a timescale ranging from milliseconds to seconds [29]. The mechanism of how TRPV1 channel desensitization occurs in MPS rats merits further investigation. In this study, the evidence we present demonstrates that silver needle thermal therapy mitigates mitochondrial damage and hyperalgesia by downregulating TRPV1 to suppress the expression of CaMKII.

## Conclusions

Our results indicated that in MPS rats, the expression levels of TRPV1, CaMKII, and CytC were increased, ATP content was decreased, and the co-localization of TRPV1 and CaMKII was augmented. Silver needle thermal therapy mitigated behavioral hyperalgesia and skeletal muscle mitochondrial damage by suppressing TRPV1, decreased the levels of CaMKII and CytC, and elevated ATP levels. These findings demonstrate a molecular crosstalk between TRPV1 and CaMKII within skeletal muscle. However, it is important to note that these observations do not exclude the involvement of other signaling molecules. Nonetheless, our study affirms that skeletal muscle TRPV1 may serve as a potential therapeutic target for myofascial pain syndrome, with this process likely occurring through the reduction of mitochondrial damage.
